# Observations on the clinical efficacy of rhaFGF combined with Vitamin B complex for patients with Severe Recurrent Aphthous Ulcer

**DOI:** 10.12669/pjms.37.7.4364

**Published:** 2021

**Authors:** Hui Sun, Jie Gao, Dan Li, Bing Li

**Affiliations:** 1 Hui Sun, Department of Stomatology, First Medical Center, Chinese PLA General Hospital, Beijing 100853, China; 2 Jie Gao, Department of Emergency, Shandong Weifang Yi Du Central Hospital, Weifang, Shandong, 262500, China; 3 Dan Li, Department of Stomatology, First Medical Center, Chinese PLA General Hospital, Beijing 100853, China; 4 Bing Li Department of Pain, First Medical Center, Chinese PLA General Hospital, Beijing 100853, China

**Keywords:** rhaFGF, vitamin B complex, severe RAU

## Abstract

**Objectives:**

To observe the therapeutic effect of rha FGF combined with vitamin B complex on severe recurrent aphthous ulcers (RAUs).

**Methods:**

Ninety patients with severe RAU (grade III and IV) admitted to Chinese PLA General Hospital from May 2018 to May 2019 were selected and divided into two groups using a random number table, 45 patients/group. Patients in the control group gargled with a mixture of vitamin B complex (250 ml of 9% normal saline +250 ml of vitamin B complex +160,000 U of gentamicin +25 ml of lidocaine) after oral cleaning; patients in the observation group gargled with a mixture of the same ratio after oral cleaning and then sprayed rhaFGF on their oral ulcers. The clinical symptoms and treatment effects of the 2 groups within 1 week of medication were compared.

**Results:**

The total effective rate of treatment was 97.78% in the observation group and 82.22% in the control group; the difference was statistically significant (P <0.05). The time to ulcer pain disappearance and eating recovery and the ulcer healing time in the observation group were significantly shorter than those in the control group (P <0.05).

**Conclusion:**

rhaFGF combined with vitamin B complex has a significant therapeutic effect for patients with severe RAU; it can relieve pain and illness faster and shorten the healing time of ulcers.

## INTRODUCTION

Recurrent aphthous ulcer (RAU) is a common oral mucosal injury with a prevalence of up to 20.0%.^1^-^2^ It clinically manifests as periodic recurring attacks, during which the patient’s speech and eating are affected by irritation and pain; especially for those with serious and frequent attacks, the quality of life is seriously affected.^3^ Currently, RAU is mainly treated symptomatically in the clinic, typically with vitamin B complex.^4^ Acidic fibroblast growth factor (aFGF) has been shown to promote the growth of wound granulation tissue and induce capillary angiogenesis^5^,promoting healing in various skin injuries, such as burn wounds and diabetic ulcers.^6^ However, its application in oral ulcers has rarely been reported. Recombinant human acidic fibroblast growth factor (Shanghai Terry Pharmaceutical Co., Ltd., rhaFGF) is an acidic, humanized fibroblast growth factor originally developed in China.^7^ In this study, the therapeutic effect on severe RAU was compared between the administration of vitamin B complex alone and rhaFGF combined with vitamin B complex to provide a basis for clinical application.

## METHODS

Ninety patients aged 20-60 years with severe RAU and admitted to our hospital from May 2018 to May 2019 were selected and divided into an observation group and a control group using a random number table, 45 patients/group. General data such as gender, age, number of ulcers, and course of disease were comparable between the 2 groups (P> 0.05). Each patient signed the ICF and was able to complete the treatment and follow-up.

### Ethical Approval

The study was approved by the Institutional Ethics Committee of First Medical Center, Chinese PLA General Hospital on May 10, 2019, and written informed consent was obtained from all participants.

### Inclusion and Exclusion Criteria

Patients with RAU of grade III and IV were included; patients with other oral mucosal diseases, systemic diseases, immune system diseases, etc. were excluded. No patient took any medicine for treating oral ulcers three months before participating in this study. No other measures were taken for this attack.

Grading for OM was assigned according to the WHO as follows: Grade 0, no abnormalities on the oral mucosa; Grade I: 1 ~ 2 ulcers of <1.0 cm on the oral mucosa, mild pain, no effect on eating; Grade II: 1 ulcer of > 1.0 cm and several small ulcers on the oral mucosa, worse pain than Grade I, and a semiliquid diet is tolerated; Grade III: 2 ulcers of > 1.0 cm and several small ulcers on the oral mucosa, obvious pain, and only a liquid diet is tolerated; Grade-IV: more than 2 ulcers and/or fusion ulcers of > 1.0 cm, severe pain and difficulty eating.

### Treatment

Control group: Patients in the control group gargled with a mixture of vitamin B complex (250 ml of 9% normal saline +250 ml of B vitamin B complex +160,000 U of gentamicin +25 ml of lidocaine) after cleaning the oral cavity with 20 ml warm saline, four times a day, 10 minutes after each meal and before going to bed; the liquid was kept in the mouth for five minutes after gargling and then spit out. Observation group: Patients sprayed rhaFGF (Guo Yao Zhun S20060102) onto the oral ulcers after administration of the treatment protocol used by the control group, four times a day; patients were asked not to drink water within 30 minutes after administration to keep the liquid in the oral cavity for as long as possible. Patients in both groups took the medicine for one week.

### Criteria of observation indexes and efficacy

Comparison of the total effective rates between the two groups of patients after treatment: Greatly effective: the ulcers completely or essentially healed within five days of medication application, and the pain and hyperemia had disappeared; Effective: the ulcers completely or basically healed within 7 days of medication application, and the pain and hyperemia had disappeared; Ineffective: the ulcers did not heal after seven days of medication, and the pain and hyperemia did not improve substantially. The total effective rate of treatment was calculated as (number of significantly effective + number of effective)/total number of patients × 100%.

Comparison of the clinical symptoms and recovery of the two groups of patients, including the time to pain disappearance and eating and speech recovery and the ulcer surface healing time after a course of treatment.

### Statistical Analysis

The data were statistically analyzed using SPSS 23.0. Enumeration data are described as the rate (%), comparison between groups was performed with the χ2 test, measurement data are described by (x¯ ± s) and were compared with the t test. P <0.05 was considered statistically significant.Table-I.

**Table I:** Comparison of general information between the 2 groups (n = 45, x¯ ± s).

*Group*	*Patients (n)*	*Gender*	*Age (x¯±s)*	*Ulcer No. (x¯±s)*	*Course (x¯±s)*

		*Male Female (x ?±s?*			
Observation group	45	24 21	43.50±8.50	2.80±0.80	3.95±1.07
Control group	45	22 23	44.70±7.60	3.10±0.77	4.26±1.24
χ^2^/t		0.18	0.71	1.81	1.27
p		0.67	0.48	0.07	0.21

## RESULTS

The total effective rate of the observation group was 97.78%, which was significantly higher than that of the control group (82.22%; χ2 = 6.05, P <0.05;.Table-II. The times to pain disappearance and eating and speech recovery and the ulcer healing time in the observation group were shorter than those in the control group, and the differences were statistically significant (P <0.05.Table-III

**Table II:** Comparison of the total treatment efficiency of the two groups of patients (%).

*Group*	*Patients (n)*	*Significantly effective (n)*	*Effective (n)*	*Ineffective (n)*	*Total efficiency (%)*
Observation group	45	37	7	1	97.78
Control group	45	31	6	8	82.22
χ2					6.05
p					0.01

**Table III:** Comparison of the recovery of symptoms between the 2 groups.

*Group*	*Patients*	*Time of pain disappearance (d)*	*Time to eating and speech recovery (d)*	*Healing time (d)*
Observation group	45	3.57±1.50	2.71±1.10	5.54±1.70
Control group	45	4.26±1.64	3.20±1.05	6.40±1.60
t		2.09	2.16	2.47
p		0.04	0.03	0.02

## DISCUSSION

Among all oral mucosal diseases, RAU was the first identified.^8^ The onset time of the disease, which is characterized as self-limited, recurrent and periodic, is not limited to any specific season.^9^ Currently, the pathogenesis of RAU is unclear, but it is thought to be related to multiple factors, such as genetics, local trauma, psychological factors, microbial infections, nutritional deficiencies, abnormal immune responses, and food and drug stimulation.^10^ Therefore, effective treatment methods have been the primary focus of research.

The vitamin B complex is composed of vitamin B1, vitamin B2, vitamin B6, nicotinamide and calcium pantothenate.^11^ Studies have confirmed that the various components of the vitamin B complex are involved in the formation of coenzymes, regulate the body’s carbohydrates, lipids, proteins, etc., protect sebaceous glands and mucosal tissues, promote mucosal repair and regeneration, inhibit the development of inflammation, accelerate vasodilation and blood circulation, improve blood supply, reduce oral mucosal reaction, and promote ulcer healing,^12^-^13^ but the therapeutic effect for severe RAU is poor.

Acidic fibroblast factor (aFGF) has an isoelectric point of 5-7, is acidic, and is mainly distributed in brain tissue and kidney.^14^ It was first isolated and purified from the bovine brain by Thomas in 1984. It can activate the autophosphorylation of the cytoplasmic tyrosine of fibroblast growth factor receptor (FGFR), promote cell division, and is an effective promotion factor for cell proliferation and differentiation.^15^-^16^ It plays an important role in promoting the body’s growth and development, repairing tissue damage, accelerating vascular regeneration, and promoting trophic nerve growth.^17^-^18^

From the results of this study, it can be seen that the total efficacy of treatment in the observation group was significantly higher than that in the control group; the times to ulcer pain disappearance and to eating recovery and the healing time were shorter than those in the control group, and the difference was statistically significant (P <0.05), indicating that the therapeutic effect of rhaFGF combined with vitamin B complex on severe RAU is better than that of vitamin B complex alone. rhaFGF is an acidic fibroblast growth factor that is completely consistent with the humanized sequence obtained by genetic engineering technology and has multiple biological activities. In early wound healing, rhaFGF actively and specifically binds to FGFR on the cell membrane near the wound, which can promote the division and proliferation of epithelial cells near the wound, induce their migration and accelerate the growth of granulation tissue.^19^ Furthermore, as a mitogen for blood vessels and other interstitial cells in vitro, rhaFGF can induce angiogenesis and promote capillary angiogenesis^20^, thereby accelerating the healing of mucosal wounds and shortening the healing time. In the later stages of wound healing, rhaFGF can directly or indirectly promote the apoptosis of fibroblasts, maintain the balance between cell proliferation and apoptosis, and prevent the formation of scar tissue. Moreover, the negatively charged rhaFGF has a strong affinity in the acidic environment of the wound surface and easily combines with the positively charged receptors on the cell membrane to present mild biological activity for an extended period of time. rhaFGF has no odor in the oral cavity and is easily accepted by the patients. When combined with vitamin B complex to treat patients with RAU, it can relieve pain and improve the condition within a short time.

### Limitations of this study

The number of subjects included in this study is limited, so the conclusions drawn may not be very convincing. In addition, there were few types of observational indicators used to evaluate efficacy in this study. We will consider to include more subjects in future studies, and evaluate the efficacy of rhaFGF combined with vitamin B complex in the treatment of severe recurrent oral ulcer from multiple perspectives.

## CONCLUSION

rhaFGF combined with vitamin B complex has a good curative effect for patients with severe RAU, is easy to use and has clinical significance.

### Authors’ Contributions:

**HS &** **JG:** Designed this study and prepared this manuscript, and are responsible and accountable for the accuracy or integrity of the work.

**BLi** Collected and analyzed clinical data.

**DL:** Significantly revised this manuscript.
